# *Scedosporium/Lomentospora* Species Induce the Production of Siderophores by *Pseudomonas aeruginosa* in a Cystic Fibrosis Mimic Environment

**DOI:** 10.3390/jof9050502

**Published:** 2023-04-22

**Authors:** Thaís P. Mello, Iuri C. Barcellos, Michaela Lackner, Marta H. Branquinha, André L. S. Santos

**Affiliations:** 1 Laboratório de Estudos Avançados de Microrganismos Emergentes e Resistentes (LEAMER), Departamento de Microbiologia Geral, Instituto de Microbiologia Paulo de Góes (IMPG), Centro de Ciências da Saúde (CCS), Universidade Federal do Rio de Janeiro (UFRJ), Rio de Janeiro 21941-902, RJ, Brazil; 2 Instituto Federal de Educação, Ciência e Tecnologia do Rio de Janeiro (IFRJ), Maracanã, Rio de Janeiro 20270-021, RJ, Brazil; 3 Institute for Hygiene and Medical Microbiology, Medical University of Innsbruck, Schöpfstrasse 41, 6020 Innsbruck, Austria; 4 Rede Micologia RJ—Fundação de Amparo à Pesquisa do Estado do Rio de Janeiro (FAPERJ), Rio de Janeiro 21941-902, RJ, Brazil

**Keywords:** *Scedosporium/Lomentospora*, *Pseudomonas aeruginosa*, cystic fibrosis, pyoverdine, pyochelin, siderophores

## Abstract

Over the last years, the interkingdom microbial interactions concerning bacteria and fungi cohabiting and/or responsible for human pathologies have been investigated. In this context, the Gram-negative bacterium *Pseudomonas aeruginosa* and fungal species belonging to the *Scedosporium/Lomentospora* genera are widespread, multidrug-resistant, emergent, opportunistic pathogens that are usually co-isolated in patients with cystic fibrosis. The available literature reports that *P. aeruginosa* can inhibit the in vitro growth of *Scedosporium/Lomentospora* species; however, the complex mechanisms behind this phenomenon are mostly unknown. In the present work, we have explored the inhibitory effect of bioactive molecules secreted by *P. aeruginosa* (3 mucoid and 3 non-mucoid strains) on *S. apiospermum* (*n* = 6 strains), *S. minutisporum* (*n* = 3), *S. aurantiacum* (*n* = 6) and *L. prolificans* (*n* = 6) under cultivation in a cystic fibrosis mimic environment. It is relevant to highlight that all bacterial and fungal strains used in the present study were recovered from cystic fibrosis patients. The growth of *Scedosporium/Lomentospora* species was negatively affected by the direct interaction with either mucoid or non-mucoid strains of *P. aeruginosa*. Moreover, the fungal growth was inhibited by the conditioned supernatants obtained from bacteria-fungi co-cultivations and by the conditioned supernatants from the bacterial pure cultures. The interaction with fungal cells induced the production of pyoverdine and pyochelin, 2 well-known siderophores, in 4/6 clinical strains of *P. aeruginosa*. The inhibitory effects of these four bacterial strains and their secreted molecules on fungal cells were partially reduced with the addition of 5-flucytosine, a classical repressor of pyoverdine and pyochelin production. In sum, our results demonstrated that distinct clinical strains of *P. aeruginosa* can behave differently towards *Scedosporium/Lomentospora* species, even when isolated from the same cystic fibrosis patient. Additionally, the production of siderophores by *P. aeruginosa* was induced when co-cultivated with *Scedosporium/Lomentospora* species, indicating competition for iron and deprivation of this essential nutrient, leading to fungal growth inhibition.

## 1. Introduction

Cystic fibrosis (CF) is one of the most common genetic lung diseases in the Caucasian population worldwide [1]. The disease affects multiple organs, but mainly the respiratory system, and it is caused by mutations in the cystic fibrosis transmembrane conductance regulator (*CFTR*) gene. *CFTR* gene codes for a chloride channel, which is partially responsible for maintaining airway homeostasis through water transport, chloride secretion, and sodium reabsorption [2]. Mutations in *CFTR* gene lead to production of a hyper concentrated mucus and, consequently, decreased mucociliary clearance, resulting in frequent microbial colonization/infection and concomitant airway inflammation [1,2,3].

During childhood, the airways of CF patients are mainly colonized by *Staphylococcus aureus* and *Haemophilus influenzae* and, with patient aging, the main microbial species changes to *Burkholderia cepacia* complex and *Pseudomonas aeruginosa* [1]. The constant bacterial infection leads to prolonged treatment with antimicrobials and/or corticosteroids, which favors the airways colonization by fungi [3]. When it comes to yeasts, *Candida* species are the most prevalent with *C. albicans* being the most frequent (>75%) [1,3]. Among filamentous fungi, *Aspergillus fumigatus* is the predominant species (varying from 10% to 57%) followed by the multidrug-resistant species belonging to the *Scedosporium/Lomentospora* genera, with *Scedosporium apiospermum* (24–57.6%) and *Scedosporium boydii* (19.3–65.4%) being the most frequent species of this fungal complex, followed by *Scedosporium aurantiacum* (6–50%), *Pseudallescheria ellipsoidea* (9.3%), *Lomentospora prolificans* (3.6%), and *Scedosporium minutisporum* (0.7–6%) [1,4,5]. 

Colonization of CF airways by *Scedosporium/Lomentospora* species starts with inhalation of conidia that germinate, forming hyphae, and developing a complex and robust biofilm-like structure [6,7]. The presence of *Scedosporium/Lomentospora* species in CF bronchi prevails for several months or years, and can evolve to bronchitis, pulmonary mycetoma, and disseminated infections [4]. The mortality rate in CF patients infected with *Scedosporium/Lomentospora* species is around 100% when dissemination to the central nervous system occurs [8]. This unacceptable rate is mainly caused by difficulties in the treatment due to the intrinsic multidrug-resistance profile of *Scedosporium/Lomentospora* species to almost all clinically available antifungal drugs [9]. In accordance, the resistance to antifungals belonging to the azole class is increased in an in vitro CF mimic environment, probably due to the transcriptional changes of enzymes involved in the synthesis of several plasma membrane components that occur in *Scedosporium/Lomentospora* cells in CF microenvironments [7,10]. 

While the historical approach to study CF pathogens have focused on one species at a time, it is now well-known that CF infections are mainly associated with dynamic polymicrobial communities, which directly influence the pathogenesis, antimicrobial susceptibility, and disease progression [11,12,13]. The interactions between bacteria and fungi in the CF context are coordinated by the balance of stimulatory and inhibitory effects. For example, *P. aeruginosa* secreted soluble factors are able to inhibit the conidial germination of *Scedosporium/Lomentospora* species, such as pyocyanin and *cis*-11-methyl-2-dodecenoic (DSF) [14,15]. By contrast, volatile organic compounds produced by *P. aeruginosa* stimulated the growth of *Scedosporium* spp., mainly in culture medium containing poor amounts of either nitrogen or sulfur sources [14]. Furthermore, antimicrobials, such as tobramycin, induced the growth of *Scedosporium* spp., indicating that certain antimicrobials directly and/or indirectly promoted fungal growth [14].

Iron is an essential nutrient for all microorganisms and its acquisition is necessary for the successful host colonization [16,17]. In order to obtain iron in the mammalian host, microorganisms produce chelators, known as siderophores, since iron is bound to hemoproteins or chelated by transferrin and lactoferrin [16,17]. *P. aeruginosa* cells are able to secrete a siderophore presenting low affinity for iron, named pyochelin (pFe = 16), and another one with high affinity for iron, pyoverdine (pFe = 27). The affinity of siderophores for Fe(III), under physiological conditions (pH 7.4), is measured by the pFe value, which is defined as the negative logarithm of the free Fe(III) in solution for total [ligand] = 10^−5^ mol/L and total [iron] = 10^−6^ mol/L. Both *P. aeruginosa* siderophores, pyochelin and pyoverdine, have essential roles during the microbial dispute for resources in the environment [17]. For example, in an environment with low iron concentration, pyoverdine is the main anti-*A. fumigatus* mechanism produced by *P. aeruginosa* cells [18]. In addition to the chelating function of pyochelin, this bioactive molecule also presents an antifungal activity through induction of either reactive oxygen species (ROS) or reactive nitrogen species (RNS) explosions inside the fungal cells [17]. 

Despite the importance of iron dispute in polymicrobial infections, nothing is known about this topic when the interaction process between filamentous fungi belonging to the *Scedosporium/Lomentospora* genera and the Gram-negative bacterium *P. aeruginosa* is taken into account. Based on these premises and with the focus to start to explore this intriguing subject, we have conducted an in vitro study about the interaction of *P. aeruginosa* with *S. apiospermum, S. minutisporum, S. aurantiacum* and *L. prolificans*, utilizing clinical isolates recovered from CF patients, under cultivation in a CF mimic environment medium, focusing on the inhibitory effects of pyoverdine and pyochelin on fungal growth.

## 2. Materials and Methods

### 2.1. Microorganisms

In the present work, all fungal isolates belonging to the *Scedosporium/Lomentospora* genera, *S. apiospermum* (*n* = 6; strain codes: 11–86, 11–87, 11–89, 11–90, 12–06 and 12–07), *S. minutisporum* (*n* = 3; strain codes: 10-27, 10-28 and P67), *S. aurantiacum* (*n* = 6; strain codes: 11-15, 11-85, 11-95, 12-01, 12-02 and 12-05), and *L. prolificans* (*n* = 6; strain codes: 11-84, 11-91, 12-18, 12-19, 12-23 and 12-24), were recovered from CF patients and kindly provided by Dr. Michaela Lackner (Medical University of Innsbruck, Austria) [7]. To obtain the conidial cells, each isolate was grown at room temperature on potato dextrose agar (PDA; Difco, Becton, Dickinson and Company, La Jolla, CA, USA). After 7 days in culture, conidia were obtained by washing the plate surface with sterile saline, then filtered using a 40-μm nylon cell strainer (BD) in order to remove hyphal fragments [19]. The conidial cells were counted in a Neubauer chamber. 

*Pseudomonas aeruginosa* strains (*n* = 6), which were kindly provided by Dr. Elizabeth de Andrade Marques and Dr. Robson Leão (Hospital Universitário Pedro Ernesto; Universidade do Estado do Rio de Janeiro—UERJ, Brazil) were isolated from three distinct CF individuals, designated as patient 1, 2 and 3 (Table 1). *P. aeruginosa* cells were cultured for 18 h and subcultured on Mueller Hinton Agar (Difco, Becton, Dickinson and Company, La Jolla, CA, USA) for additional 24 h at 37 °C. Subsequently, the bacterial cultures were diluted to a working solution with approximately 10^8^ colony-forming units (CFU) per mL [20]. 

### 2.2. Co-Culture of Scedosporium/Lomentospora Species and P. aeruginosa

To analyze the direct effect of *P. aeruginosa* cells on planktonic growth of *Scedosporium/Lomentospora* species, 10^6^ conidia of each fungal isolate were jointed with 10^6^ CFUs of each *P. aeruginosa* strain (1:1 ratio) in a 96-well plate containing synthetic cystic fibrosis sputum medium (SCFM) [21]. After incubation for 24 h at 37 °C in an atmosphere containing 5% CO_2_, the polysaccharide chitin in the fungal cell wall was stained with 5 µg/mL of Calcofluor white (Sigma-Aldrich, St. Louis, MO, USA) for 1 h at room temperature. The fluorescence intensities were measured using a SpectraMax Gemini XPS Fluorescence Microplate Reader (Molecular Devices, Sunnyvale, CA, USA) at an excitation wavelength of 355 nm and an emission wavelength of 430 nm [22,23]. 

To analyze the effects of *P. aeruginosa* on fungal biofilms, *Scedosporium/Lomentospora* conidial suspensions in SCFM (200 µL containing 10^6^ cells) were placed on flat-bottom 96-well polystyrene microtiter plates and then incubated at 37 °C in an atmosphere with 5% CO_2_ for 24 h to permit the biofilm formation (designate as 24 h mature fungal biofilm) [19]. Subsequently, *P. aeruginosa* cells were added to the 24 h mature fungal biofilms systems. 

*S. apiospermum* (strain 12-07), *S. minutisporum* (strain 10-28), *S. aurantiacum* (strain 11-15) and *L. prolificans* (strain 12-19) were randomly selected for all subsequent experiments. 

### 2.3. Pyoverdine and Pyochelin Measurements 

After 24 h of bacteria-fungi co-cultivation, as described in Section 2.2, the conditioned supernatants were collected, centrifuged (30 min at 15,000 rpm), and filtered through a 0.22-µm membrane (Millipore, São Paulo, SP, Brazil) in order to withdraw remaining cells. Then, aliquots (100 µL) of these sterilized supernatants were transferred to a black 96-well plate and fluorescence was measured with excitation/emission of 405/455 nm for pyoverdine and 360/460 nm for pyochelin in a SpectraMax Gemini XPS Fluorescence Microplate Reader (Molecular Devices, Sunnyvale, CA, USA) [20,24].

### 2.4. Influence of Direct Contact between Fungi and Bacteria on Pyoverdine and Pyochelin Production

To determine whether the direct contact between *P. aeruginosa* and *Scedosporium/Lomentospora* cells is required to modulate the pyoverdine and pyochelin production, Nunc™ Cell Culture Inserts in Carrier Plate Systems assay (ThermoFisher Scientific, Waltham, MA, USA) was employed. In this experiment, bacteria and fungi had the growth physically separated but the same culture medium was shared. To perform this, inoculums of 1.5 mL containing *Scedosporium/Lomentospora* (5 × 10^6^ conidia/mL in SCFM) were added to the upper chamber and the lower chamber were inoculated with 1.5 mL bacterial cells (5 × 10^6^ CFU/mL in SCFM). The systems were incubated for 24 h at 37 °C in an atmosphere with 5% CO_2_, then the siderophores produced by *P. aeruginosa* cells were measured as previously described in Section 2.3.

### 2.5. Effect of Fungal Metabolism on Pyoverdine and Pyochelin Production

In these experiments, the *Scedosporium/Lomentospora* conidia were fixed with 4% paraformaldehyde at 4 °C for 30 min [19] before the co-incubation with *P. aeruginosa* cells, as detailed in Section 2.2, in order to ascertain whether pyoverdine and pyochelin production is correlated to the active competition for available nutrients. After 24 h of interaction, *P. aeruginosa* siderophores were measured as previously described in Section 2.3.

### 2.6. Effect of Iron Concentration on Pyoverdine and Pyochelin Production

To evaluate the influence of iron concentration in siderophores production, the bacteria-fungi co-cultures were performed as previously described in SCFM, containing either 3.6 µM (standard concentration) or 36 μM FeSO_4_. Then, *P. aeruginosa* siderophores production was measured as described in Section 2.3. 

### 2.7. Effect of P. aeruginosa Secreted Molecules on Fungal Growth

Conditioned supernatants obtained from either bacteria-fungi co-cultivation (as previously described in Section 2.2 and Section 2.3) or from *P. aeruginosa* pure cultures were tested in this set of experiments. Posteriorly to sterilization of conditioned supernatants, 10^6^ conidia or 24 h-mature fungal biofilms (formed as previously described in Section 2.2) were incubated with 50% of conditioned supernatants in SCFM for 24 h at 37 °C in an atmosphere with 5% CO_2_. As controls, 100% SCFM and 50% SCFM plus 50% saline (0.85% NaCl) were utilized, in order to evaluate the role of nutrient restriction in fungal inhibition [25]. Then, the fungal biomass was assessed with Calcofluor white as previously described in Section 2.2. 

### 2.8. Effect of 5-Flucytosine on Pyoverdine and Pyochelin Production

*P. aeruginosa* growth in pure cultures and the bacteria-fungi co-cultivations (as previously described in Section 2.2) were performed in the absence and in the presence of 10 μM 5-flucytosine (5-FC; Sigma-Aldrich, St. Louis, MO, USA), in order to inhibit the pyoverdine and pyochelin production [26]. Then, *P. aeruginosa* siderophores production was measured as previously described in Section 2.3. 

### 2.9. Role of Pyoverdine and Pyochelin on Fungal Growth 

The effects of conditioned supernatants obtained in the presence of 5-FC (Section 2.8) on fungal growth were also determined. As control, the effect of 5-FC (10 μM) alone on the fungal growth was also evaluated.

### 2.10. Statistics

All experiments were performed in triplicate, in three independent experimental sets. Data were expressed as mean ± standard deviation (SD). The results were evaluated by ANOVA test followed by Dunnett’s multiple comparison test using GraphPad Prism 8 computer software (GraphPad Software, Inc., La Jolla, CA, USA). In all analyses, *p*-values of 0.05 or less were considered statistically significant.

## 3. Results

### 3.1. P. aeruginosa Cells Inhibit the Growth of Scedosporium/Lomentospora Species

**Figure 1:**
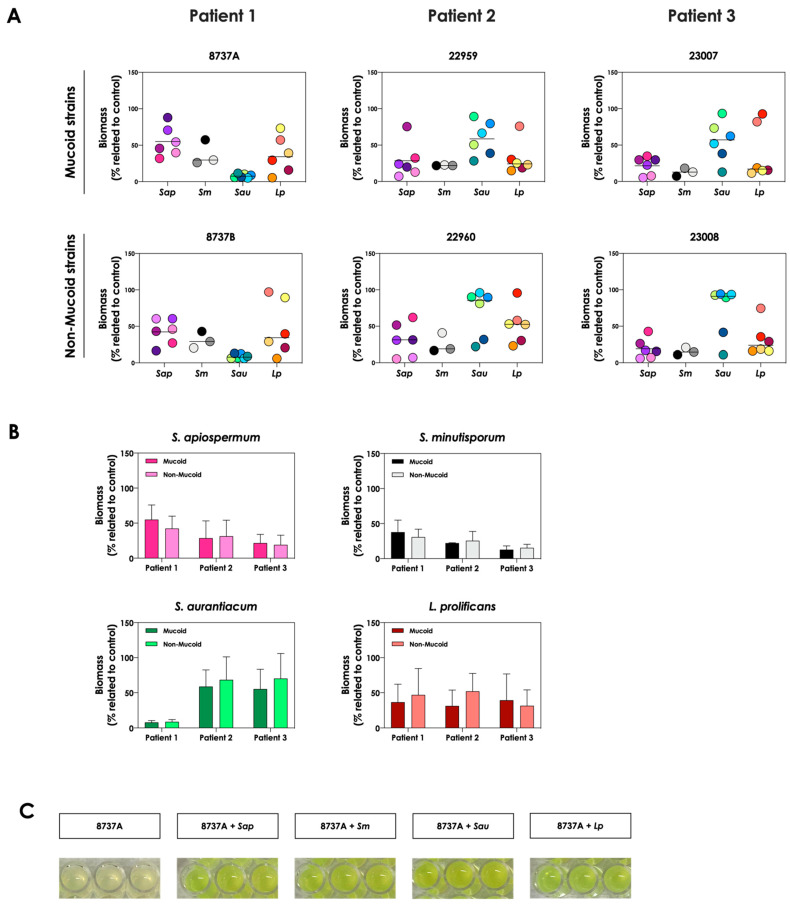
Growth analysis of distinct CF clinical strains of *S. apiospermum* (*Sap*; strains 11-86, 11-87, 11-89, 11-90, 12-06 and 12-07), *S. minutisporum* (*Sm*; strains10-27, 10-28 and P67), *S. aurantiacum* (*Sau*; strains 11-15, 11-85, 11-95, 12-01, 12-02 and 12-05), and *L. prolificans* (*Lp*; strains 11-84, 11-91, 12-18, 12-19, 12-23 and 12-24) when cultivated in the absence (control) and in the presence of mucoid (8737A, 22959 and 23007) or non-mucoid (8737B, 22960 and 23008) *P. aeruginosa* strains. (**A**) Fungi (10^6^ conidial cells) were placed to interact with *P. aeruginosa* strains during 24 h at 37 °C in SCFM. Then, the systems were processed to detect the fungal growth by labeling the chitin present in the fungal cell wall with Calcofluor white. The results are expressed as the percentage of fungal growth (biomass) in the presence of *P. aeruginosa* in relation to the control (fungal growth in SCFM alone). Each fungal isolate is represented by a colorful circle. The black lines indicate the mean growth for each fungal species in the presence of *P. aeruginosa.* (**B**) Comparison of the fungal growth in the presence of mucoid and non-mucoid *P. aeruginosa* strains isolated from the same patient. The results are expressed as the growth mean of all fungal strains from *S. apiospermum, S. minutisporum, S. aurantiacum* and *L. prolificans* in the presence of mucoid or non-mucoid *P. aeruginosa* strains. (**C**) A representative result showing the differences observed in the color of conditioned supernatants obtained from *P. aeruginosa* (strain 8737A) in pure culture or in co-culture with *Scedosporium/Lomentospora* species.

First, the growth dynamic of *S. apiospermum* (*n* = 6)*, S. minutisporum* (*n* = 3)*, S. aurantiacum* (*n* = 6) and *L. prolificans* (*n* = 6) was investigated when co-cultured in the presence of mucoid (*n* = 3) or non-mucoid (*n* = 3) strains of *P. aeruginosa* for 24 h at 37 °C at 5% CO_2_ in a cystic fibrosis environment (SCFM). Under these experimental conditions, the growth of all 21 fungal isolates was impaired, at different degrees, by all 6 clinical strains of *P. aeruginosa* (Figure 1A). In general, the growth of *S. apiospermum* in relation to the control ranged from 5.3% to 87.9%, *S. minutisporum* from 7.3% to 57.5%, *S. aurantiacum* from 5.9% to 96%, and *L. prolificans* from 5.5% to 97.1% (Figure 1A), respectively. No significant difference on the fungal inhibitory ability was detected between mucoid and non-mucoid bacterial strains isolated from the same patient (Figure 1B). After the co-cultivation of fungal-bacterial strains, it was noticed that the color of the conditioned supernatants changed to a brighter yellowish/greenish, as mainly observed in the *P. aeruginosa* strain 8737A, when compared to the conditioned supernatants derived from the bacterial pure cultures (Figure 1C). So, this phenomenon was further investigated.

### 3.2. Pyoverdine and Pyochelin Production Is Augmented in Co-Culture of P. aeruginosa-Scedosporium/Lomentospora Species 

**Figure 2:**
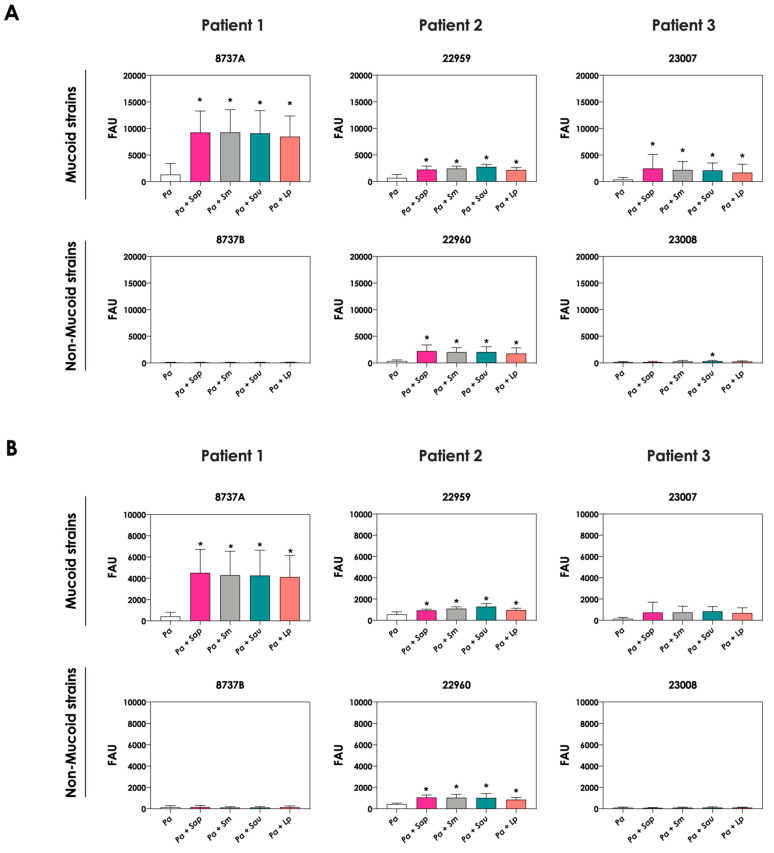
Analysis of pyoverdine and pyochelin concentration in conditioned supernatants of *P. aeruginosa* (*Pa*; mucoid strains 8737A, 22959 and 23007, and non-mucoid strains 8737B, 22960 and 23008) or conditioned supernatants obtained from bacteria-fungi interactions [*Pa* + *Sap* (*S. apiospermum*); *Pa* + *Sm* (*S. minutisporum*); *Pa* + *Sau* (*S. aurantiacum*); *Pa* + *Lp* (*L. prolificans*)]. Conditioned supernatants from 24 h of growth in SCFM were collected, filtered and the fluorescence was measured with excitation/emission of 405/455 nm for (**A**) pyoverdine and 360/460 nm for (**B**) pyochelin. The results were expressed as fluorescent arbitrary units (FAU). The asterisks (*) denote significant differences between the amount of pyoverdine/pyochelin produced by *P. aeruginosa* pure culture and *P. aeruginosa* in co-culture with *Scedosporium/Lomentospora* conidia (*p* < 0.05; 2way ANOVA, Dunnett’s multiple comparison test).

The change in the color of conditioned supernatant was previously reported in co-cultures of *C. albicans* yeasts with *P. aeruginosa* cells, and this phenomenon was associated with the increase in pyoverdine concentration in the extracellular milieu [20]. In order to check the same effect in the co-cultivation of *P. aeruginosa* and *Scedosporium/Lomentospora* species, the productions of pyoverdine and a second siderophore secreted by *P. aeruginosa*, pyochelin, were measured. The concentrations of pyoverdine in the conditioned supernatant were 3- to 6-fold higher when the *P. aeruginosa* strains 8737A, 22959, 22960 and 23007 were co-cultured with *Scedosporium/Lomentospora* conidia in comparison with the bacterial pure cultures (Figure 2A). For pyochelin (Figure 2B), an increase of 2- to 10-times in its production was detected in conditioned supernatants derived from bacteria-fungi cultivation compared to bacterial pure cultures. In summary, with the exception of one non-mucoid bacterial strain (designated as 22960), the induction of siderophore production by fungal cells occurred in mucoid *P. aeruginosa* strains. 

**Figure 3:**
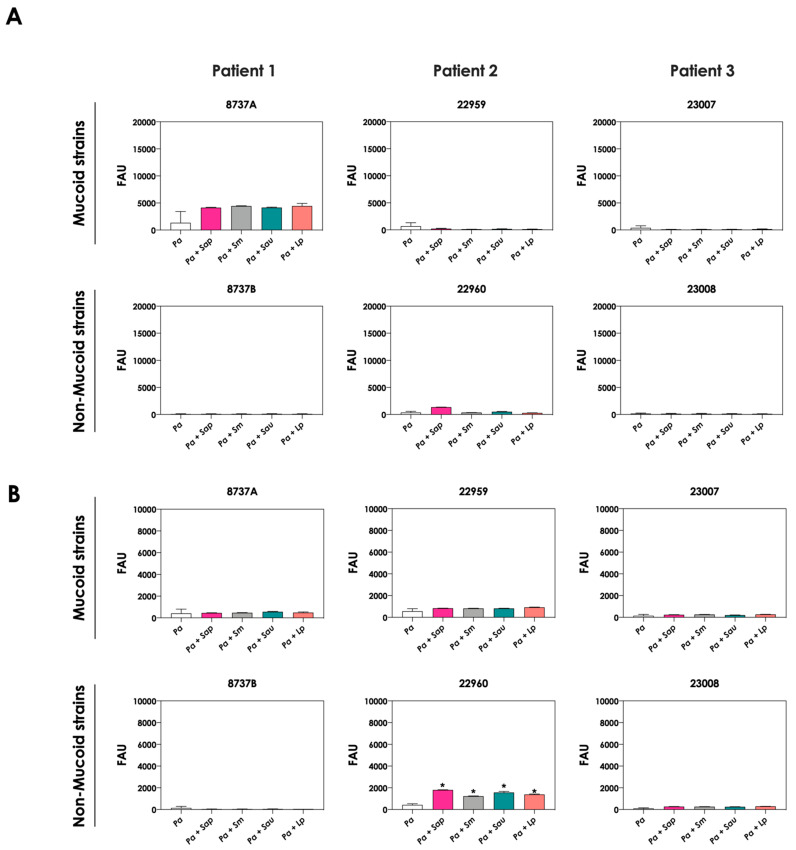
Analysis of pyoverdine and pyochelin production by *P. aeruginosa* strains pure cultures (*Pa*; mucoid strains 8737A, 22959 and 23007; and non-mucoid strains 8737B, 22960 and 23008) and *P. aeruginosa* strains in co-culture with 24 h-mature fungal biofilms [*Pa* + *Sap* (*S. apiospermum*); *Pa* + *Sm* (*S. minutisporum*); *Pa* + *Sau* (*S. aurantiacum*); *Pa* + *Lp* (*L. prolificans*)]. The conditioned supernatants were collected after 24 h, then filtered, and the fluorescence was measured with excitation/emission of 405/455 nm for (**A**) pyoverdine and 360/460 nm for (**B**) pyochelin. The results were expressed as fluorescent arbitrary units (FAU). The asterisks (*) denote significant differences between the amount of pyoverdine/pyochelin produced by *P. aeruginosa* pure culture and *P. aeruginosa* in co-culture with *Scedosporium/Lomentospora* mature biofilms (*p* < 0.05; 2-way ANOVA, Dunnett’s multiple comparison test).

The production of siderophores by *P. aeruginosa* was also evaluated after the interaction with mature biofilms of *Scedosporium/Lomentospora* species. In general, no significant increase in both pyoverdine and pyochelin concentrations were detected in the conditioned supernatants recovered from the interaction of *P. aeruginosa* cells with 24 h mature biofilms formed by *Scedosporium/Lomentospora* species, except for the pyochelin production measured in the fungi-*P. aeruginosa* strain 22960 co-cultivations (Figure 3). 

### 3.3. Bacteria-Fungi Contact Is Not Necessary for Stimulation of Pyoverdine and Pyochelin Production

**Figure 4:**
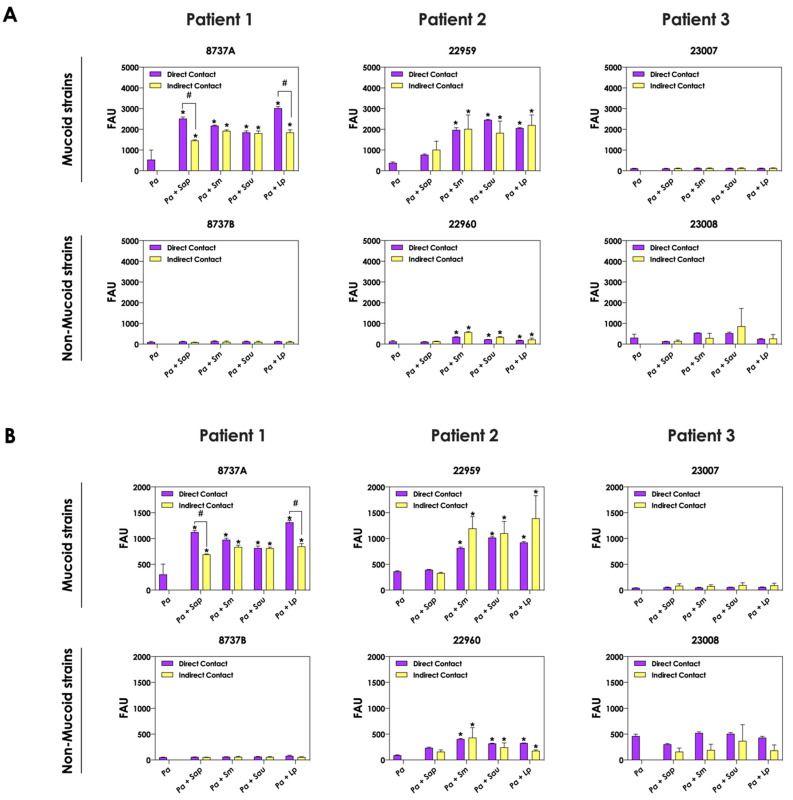
Analysis of pyoverdine and pyochelin production by *P. aeruginosa* when grown in pure culture (*Pa*; mucoid strains 8737A, 22959 and 23007; and non-mucoid strains 8737B, 22960 and 23008) or sharing the same culture medium (SCFM), although physically separated by a membrane, with *Scedosporium/Lomentospora* species [*Pa* + *Sap* (*S. apiospermum*); *Pa* + *Sm* (*S. minutisporum*); *Pa* + *Sau* (*S. aurantiacum*); *Pa* + *Lp* (*L. prolificans*)]. The conditioned supernatants after 24 h of growth were collected, filtered and the fluorescence was measured with excitation/emission of 405/455 nm for (**A**) pyoverdine and 360/460 nm for (**B**) pyochelin. The results were expressed as fluorescent arbitrary units (FAU). The asterisks (*) denote significant differences between the amount of pyoverdine/pyochelin produced by *P. aeruginosa* pure culture and *P. aeruginosa* in co-culture with *Scedosporium/Lomentospora* conidia, and the symbol # indicates significant differences between the production of siderophores in co-cultures with direct contact or not (*p* < 0.05; 2-way ANOVA, Dunnett’s multiple comparison test).

The role of direct cell–cell interactions in the induction of pyoverdine and pyochelin production by *P. aeruginosa* was evaluated. For this purpose, bacterial and fungal cells were cultured physically separated, but sharing the same culture medium (SFCM). In general, the concentrations of pyoverdine (Figure 4A) and pyochelin (Figure 4B) were the same considering the direct contact or not between *P. aeruginosa* and *Scedosporium/Lomentospora* cells. A significant reduction in the production of both bacterial siderophores was only detected in the indirect contact of *P. aeruginosa* 8737A strain co-cultured with *S. apiospermum* and *L. prolificans* when compared to the direct contact (Figure 4A,B).

### 3.4. Viable Fungi Are Necessary to Induce the Production of Siderophores by P. aeruginosa 

**Figure 5:**
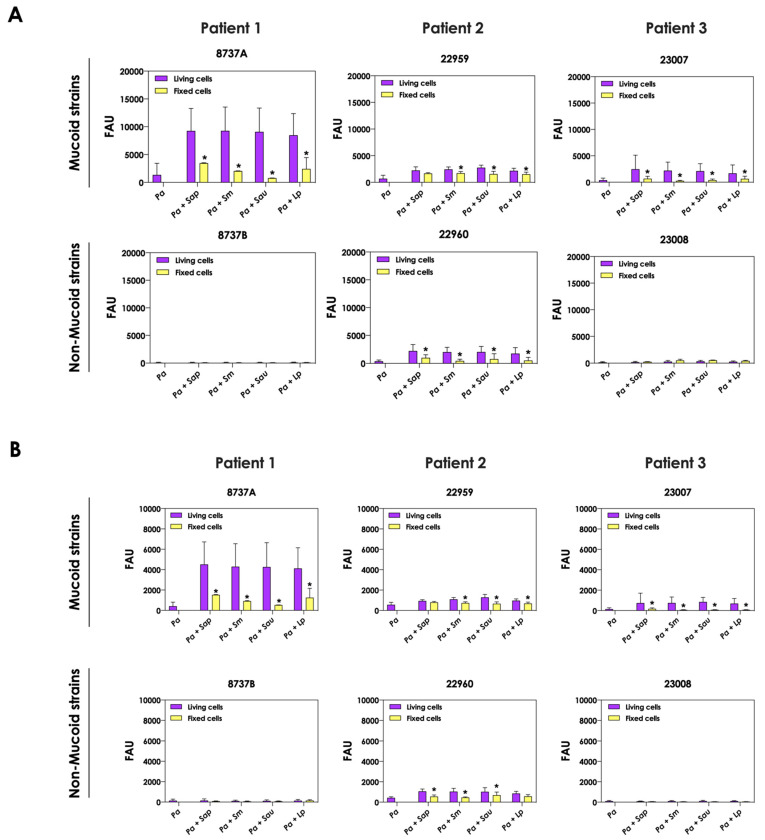
Analysis of pyoverdine and pyochelin production by *P. aeruginosa* in pure culture (*Pa*; mucoid strains 8737A, 22959 and 23007; and non-mucoid strains 8737B, 22960 and 23008) and *P. aeruginosa* in co-culture with fixed conidial cells of *Scedosporium/Lomentospora* species [*Pa* + *Sap* (*S. apiospermum*); *Pa* + *Sm* (*S. minutisporum*); *Pa* + *Sau* (*S. aurantiacum*); *Pa* + *Lp* (*L. prolificans*)]. The conditioned supernatants obtained after 24 h of growth in SCFM were collected, filtered and the fluorescence was measured with excitation/emission of 405/455 nm for (**A**) pyoverdine and 360/460 nm for (**B**) pyochelin. The results were expressed as fluorescent arbitrary units (FAU). The asterisks (*) denote significant differences between the amount of pyoverdine/pyochelin produced by *P. aeruginosa* in co-culture with living or fixed *Scedosporium/Lomentospora* conidial cells (*p* < 0.05; 2-way ANOVA, Dunnett’s multiple comparison test).

Next, we evaluated the production of pyoverdine and pyochelin by bacterial cells in co-culture with non-viable, metabolically inactivated *Scedosporium/Lomentospora* conidia. The concentrations of pyoverdine (Figure 5A) and pyochelin (Figure 5B) were drastically reduced during the contact of living *P. aeruginosa* cells and fixed fungi, reaching the same levels when bacterial cells were cultivated as pure cultures. These results clearly demonstrated that alive fungal cells, with active metabolism, are necessary to stimulate the production of both pyoverdine and pyochelin molecules by *P. aeruginosa* cells. 

### 3.5. Iron Modulates the Production of Pyoverdine and Pyochelin by P. aeruginosa in Co-Culture with Scedosporium/Lomentospora 

**Figure 6:**
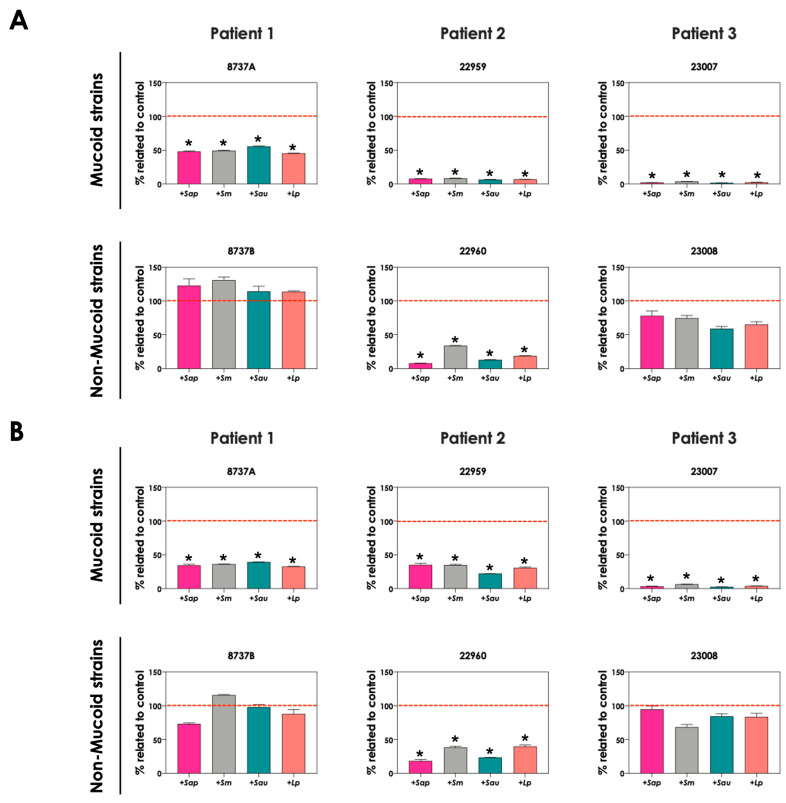
Analysis of pyoverdine and pyochelin production by *P. aeruginosa* (mucoid strains 8737A, 22959 and 23007; and non-mucoid strains 8737B, 22960 and 23008) in co-culture with *S. apiospermum* (+*Sap*), *S. minutsiporum* (+*Sm*), *S. aurantiacum* (+*Sau*) or *L. prolificans* (+*Lp*) conidial cells in SCFM containing different iron concentrations. The conditioned supernatants obtained from 24 h of growth in SCFM with either 3.6 μM or 36 μM of FeSO_4_ were collected, filtered, and the fluorescence was measured with excitation/emission of 405/455 nm for (**A**) pyoverdine and 360/460 nm for (**B**) pyochelin. The results were expressed as percentage of siderophore production obtained in 36 μM FeSO_4_ in comparison to the production at 3.6 μM (which is the original SCFM composition, here named as control and highlighted by the red dashed line). The asterisks (*) denote significant differences between the amount of pyoverdine/pyochelin produced by *P. aeruginosa* in SCFM containing 3.6 μM or 36 μM of FeSO_4_ (*p* < 0.05; 2-way ANOVA, Dunnett’s multiple comparison test).

The production of pyoverdine and pyochelin in SCFM containing two different iron concentrations (3.6 μM, which is the standard concentration in SCFM [21], and 36 μM FeSO_4_, a 10-fold higher concentration) were evaluated. The production of pyoverdine (Figure 6A) and pyochelin (Figure 6B) in the presence of *S. apiospermum, S. minutisporum, S. aurantiacum* and *L. prolificans* were significantly lower in an environment containing 36 μM FeSO_4_ than in 3.6 μM for *P. aeruginosa* strains 8737A, 22959, 22960 and 23007. The results demonstrated that *Scedosporium* and *Lomentospora* species were able to induce the production of both pyoverdine and pyochelin by *P. aeruginosa* cells, mainly in an environment with low iron concentration, which is in accordance with the necessity of competition for this nutrient when its concentration is limited. 

### 3.6. P. aeruginosa Cells Secrete Molecules Partially Responsible for Scedosporium/Lomentospora Growth Inhibition

**Figure 7:**
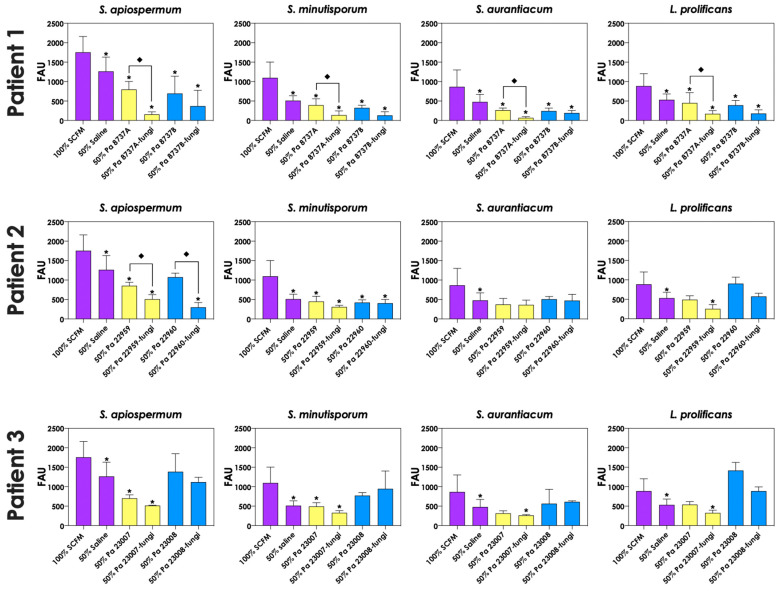
Effects of conditioned supernatants of *P. aeruginosa* on *Scedosporium/Lomentospora* growth. The conditioned supernatants obtained from *P. aeruginosa* in monoculture (i.e., Pa 8737A, Pa 8737B, Pa 22959, Pa 22960, Pa 23007 or Pa 23008) and *P. aeruginosa* grown in co-culture with *Scedosporium/Lomentospora* species (i.e., Pa 8737A-fungi, Pa 8737B-fungi, Pa 22959-fungi, Pa 22960-fungi, Pa 23007-fungi or Pa 23008-fungi) were collected, filtered, and diluted by 50% (*v*/*v*) in SCFM and, subsequently, incubated with *Scedosporium/Lomentospora* species (10^6^ conidia) at 37 °C for 24 h. Systems were processed to detect the fungal growth by labeling the chitin present in fungal cell wall with Calcofluor white. The results were expressed as fluorescent arbitrary units (FAU). The systems “50% saline” were utilized as controls of nutrients’ restrictions. The asterisks (*) denote significant differences between the fungal growth in 100% SCFM and the other systems. The diamonds (**◆**) denote significant differences between the effect of conditioned supernatants obtained from *P. aeruginosa* monoculture and conditioned supernatants from *P. aeruginosa-Scedosporium/Lomentospora* co-culture on fungal growth (*p* < 0.05; 2-way ANOVA, Dunnett’s multiple comparison test).

**Figure 8:**
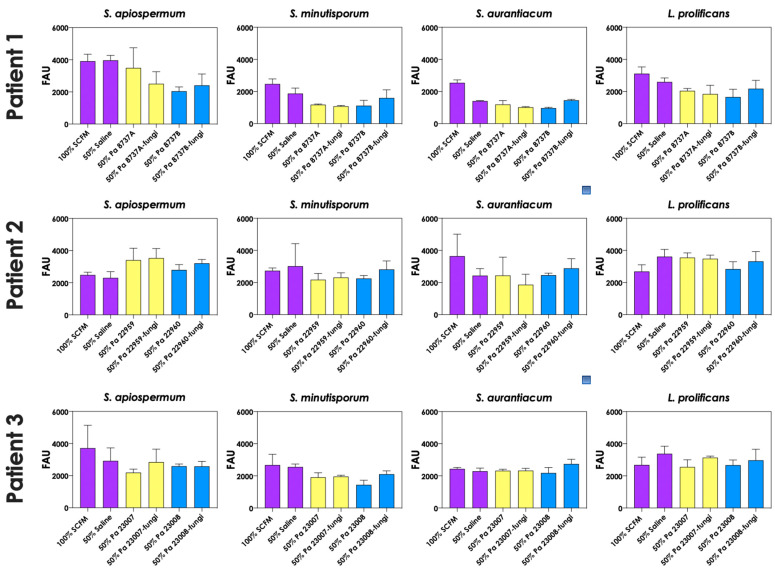
The conditioned supernatants obtained from *P. aeruginosa* in monoculture (i.e., Pa 8737A, Pa 8737B, Pa 22959, Pa 22960, Pa 23007, or Pa 23008) and *P. aeruginosa* grown in co-culture with *Scedosporium/Lomentospora* species (i.e., Pa 8737A-fungi, Pa 8737B-fungi, Pa 22959-fungi, Pa 22960-fungi, Pa 23007-fungi, or Pa 23008-fungi) were collected, filtered, and diluted by 50% (*v*/*v*) in SCFM and, subsequently, incubated with 24 h mature biofilms formed by *Scedosporium/Lomentospora* at 37 °C for 24 h. Systems were processed to detect the fungal growth by labeling the chitin present in fungal cell wall with Calcofluor white. The results were expressed as fluorescent arbitrary units (FAU). The systems “50% saline” were utilized as controls of nutrients’ restrictions.

To test whether pyoverdine and pyochelin molecules contribute to the inhibition of *Scedosporium/Lomentospora* growth, conidial cells (Figure 7) and 24 h-mature fungal biofilms (Figure 8) were incubated with 50% of conditioned supernatants obtained from either bacteria-fungi co-cultivation or *P. aeruginosa* in pure culture. Incubation of *Scedosporium/Lomentospora* conidia in SCFM containing 50% (*v*/*v*) of both test conditioned supernatants impaired the fungal growth. However, the inhibitory effect of the conditioned supernatant from bacterial-fungi co-cultivation was usually superior compared to the conditioned supernatant obtained from the axenic culture of *P. aeruginosa* cells (Figure 7). These results are in accordance with the observed increase in pyoverdine and pyochelin concentrations in conditioned supernatants of *P. aeruginosa* strains 8737A, 22959, 22960 and 23007 when cultivated in the presence of *Scedosporium/Lomentospora* cells. In contrast, no inhibitory effect was detected when 24 h-mature fungal biofilms were incubated with both test conditioned supernatants (Figure 8). Altogether, these results indicate that the effect of deprivation of iron in fungal cells caused by *P. aeruginosa* siderophores is more prominent in *Scedosporium/Lomentospora* conidia than in mature biofilms.

### 3.7. Pyoverdine and Pyochelin Are Partially Responsible for Scedosporium/Lomentospora Growth Inhibition

**Figure 9:**
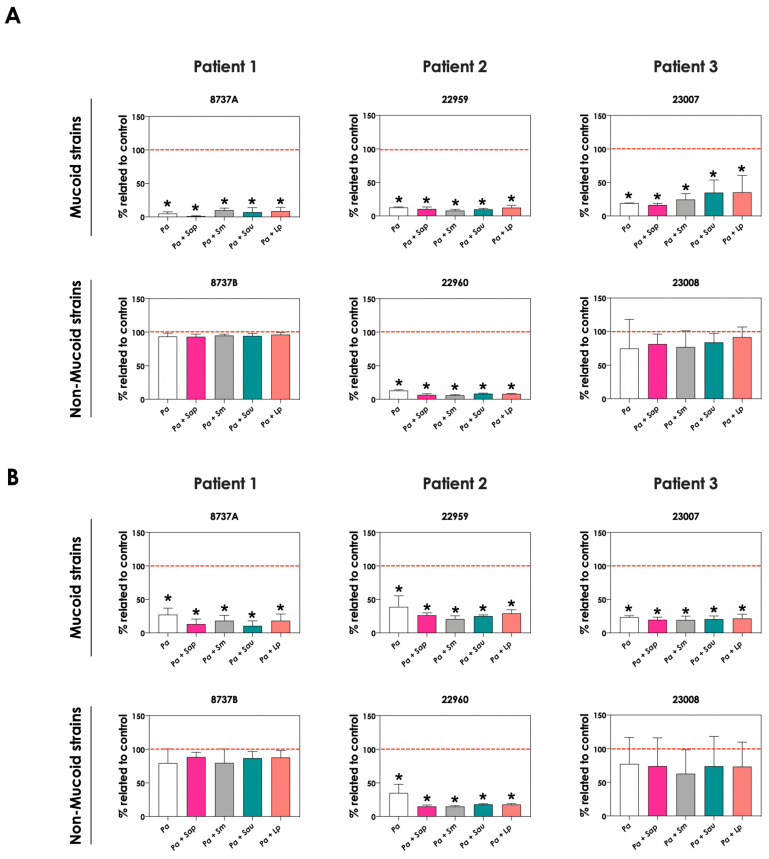
Effects of 5-flucytosine (5-FC) on pyoverdine and pyochelin production by *P. aeruginosa* in pure culture (*Pa*; mucoid strains 8737A, 22959 and 23007; and non-mucoid strains 8737B, 22960 and 23008) and in co-culture with *Scedosporium/Lomentospora* species [*Pa* + *Sap* (*S. apiospermum*); *Pa* + *Sm* (*S. minutisporum*); *Pa* + *Sau* (*S. aurantiacum*); *Pa* + *Lp* (*L. prolificans*)]. The conditioned supernatants were obtained after 24 h of growth in SCFM, then they were collected, filtered, and the fluorescence was measured with excitation/emission of 405/455 nm for (**A**) pyoverdine and 360/460 nm for (**B**) pyochelin. Results are expressed as percentage of siderophore production obtained in the presence of 5-FC in relation to the absence of this antifungal (control, highlighted by the red dashed line). The asterisks (*) denote significant differences between the amount of pyoverdine/pyochelin produced by *P. aeruginosa* in the presence or not of 5-FC (*p* < 0.05; 2-way ANOVA, Dunnett’s multiple comparison test).

**Figure 10:**
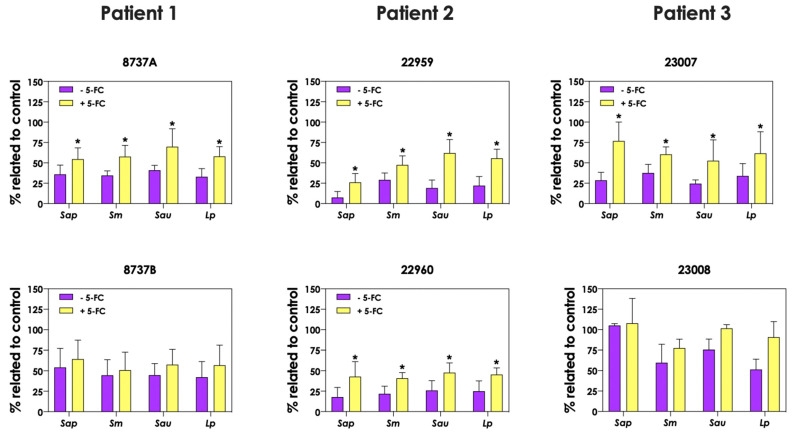
Analysis of *Scedosporium/Lomentospora* growth after incubation with conditioned supernatants obtained from *P. aeruginosa* (mucoid strains 8737A, 22959 and 23007; and non-mucoid strains 8737B, 22960 and 23008) grown in the presence or not of 10 μM 5-flucytosine (5-FC). Conidia (10^6^) of *S. apiospermum* (*Sap*), *S. minutisporum* (*Sm*), *S. aurantiacum* (*Sau*) and *L. prolificans* (*Lp*) were incubated in 50% (*v*/*v*) conditioned supernatants of *P. aeruginosa* for 24 h at 37 °C. Then, the systems were processed to detect the fungal growth by labeling the chitin present in fungal cell walls with Calcofluor white. Results are expressed as percentage of fungal growth after incubation in conditioned supernatants of *P. aeruginosa* in relation to fungal growth in 100% SCFM (control). The asterisks (*) denote significant differences between the fungal growth in conditioned supernatant of *P. aeruginosa* obtained in the presence or in the absence of 5-FC (*p* < 0.05; 2-way ANOVA, Dunnett’s multiple comparison test).

In order to evaluate the role of pyoverdine and pyochelin in fungal growth inhibition, 5-FC was used. First, the concentrations of both pyoverdine (Figure 9A) and pyochelin (Figure 9B) secreted by *P. aeruginosa* into the extracellular milieu were analyzed when the bacterial cells were incubated in the presence of 10 μM 5-FC. The results showed that the production of both siderophores was inhibited by 5-FC in the following *P. aeruginosa* strains: 8737A, 22959, 22960 and 23007. The concentrations of pyoverdine produced by *P. aeruginosa* cells were approximately 93.3%, 89.8%, 92.8% and 72.2% lower for these strains, respectively, when grown in the presence of 5-FC compared to the absence of this antifungal (Figure 9A). Similar results were observed for pyochelin measurements, in which the inhibition levels were around 85.0%, 74.7%, 83.6% and 79.8% for strains 8737A, 22959, 22960 and 23007, respectively (Figure 9B). In accordance, conditioned supernatants of these four *P. aeruginosa* strains obtained after growth in the presence of 10 μM 5-FC presented significant less antifungal effect on *S. apiospermum, S. minutisporum, S. aurantiacum* and *L. prolificans* (Figure 10), proving the role of *P. aeruginosa* siderophores in *Scedosporium/Lomentospora* growth inhibition. Pertinent to emphasize that the growth of *Scedosporium/Lomentospora* cells were not influenced by the presence of 10 μM of 5-FC.

## 4. Discussion

In this study, we evaluated the in vitro interaction between *P. aeruginosa* and *Scedosporium/Lomentospora* species (*S. apiospermum, S. minutisporum, S. aurantiacum* and *L. prolificans*) using clinical isolates recovered from CF patients. For this purpose, SCFM, a liquid culture medium that mimics the CF sputum, was employed. Our results demonstrated that all *P. aeruginosa* strains inhibited the growth of different isolates of *S. apiospermum, S. minutisporum, S. aurantiacum* and *L. prolificans* after 24 h of interaction, which is in agreement with previous in vitro studies focusing on the co-culture of *P. aeruginosa* and *Scedosporium/Lomentospora* spp. [14,15,26,27,28,29]. However, we did not observe differences in the ability of mucoid and non-mucoid *P. aeruginosa* strains to inhibit the fungal growth. In this sense, the results presented herein are in contrast to a previous study using both *S. aurantiacum* and *L. prolificans*, in which non-mucoid strains of *P. aeruginosa* inhibited more effectively the fungal growth compared to mucoid bacterial counterparts [29]. The divergences in the results should be due to the differences in the methodology employed in each study, mainly the culture media (liquid SCFM vs. Sabouraud dextrose agar), fungal growth evaluation methods (Calcofluor white vs. XTT reduction assay), besides the different *P. aeruginosa* strains [29]. Although some of the in vitro studies have demonstrated a negative interaction between *Scedosporium/Lomentospora* and *P. aeruginosa*, the data of CF patients in Germany showed a positive association regarding the isolation of these microorganisms [30]. Thus, it is important to highlight that in CF patients the interactions between *P. aeruginosa* and *Scedosporium/Lomentospora* are also influenced by other microorganisms of the lung microbiome, use of antibiotics and corticosteroids, among other factors that are not usually evaluated altogether in in vitro studies [13,14,31]. In fact, the in vitro growth of *Scedosporium* species is most unaffected by the presence of corticosteroid compounds (i.e., hydrocortisone, prednisone and methylprednisolone); whereas the antimicrobial tobramycin can stimulate its growth [14]. The interaction between *Scedosporium* and *P. aeruginosa* is a novelty and controversial topic, with conflicting results being reported taking into account both in vitro and in vivo approaches. 

Interestingly, *P. aeruginosa* cells are able to inhibit the growth of other fungal species, such as *A. fumigatus*, *Cryptococcus neoformans*, *C. albicans*, *Rhizopus microsporus,* and *Trichophyton* spp. [18,32,33,34,35,36]. The fungal growth inhibition occurs mainly due to the bacterial secreted molecules, such as phenazines (e.g., pyocyanin, 1-hydroxyphenazine), quorum-sensing compounds (e.g., acyl-homoserine lactones and alkyl quinolones), dirhamnolipids and siderophores (e.g., pyoverdine and pyochelin) [25,37,38,39]. Studies concerning the inhibitory effect of molecules produced by *P. aeruginosa* on fungal growth are mainly performed with purified commercial compounds and/or *P. aeruginosa* pure culture supernatant [14,15,28,34,36,40,41]; the studies with molecules produced in co-culture with fungi are scarce [20,42]. Nevertheless, the interaction between microorganisms leads to a differential production of extracellular molecules [20,42]. For example, the interaction between *P. aeruginosa* and *A. fumigatus* induced the production of bacterial (e.g., rhamnolipids and different analogs of pyoverdine) and fungal (e.g., gliotoxin) secondary metabolites [42]. Similarly, the in vitro interactions between *P. aeruginosa* and *C. albicans,* or *P. aeruginosa* and *R. microsporus* induced the production of pyoverdines [20,25]. Conversely, in a mixed infection in neutropenic mouse model, *C. albicans* cells secreted proteins that suppress pyoverdine and pyochelin gene expression [43]. In this context, herein, we utilized conditioned supernatants produced in mixed cultures to evaluate the role of *P. aeruginosa* secreted molecules in *Scedosporium/Lomentospora* growth inhibition. 

Due to the changes in the color of the conditioned supernatant obtained from bacteria-fungi co-cultures and the important role of siderophores in inter-kingdom interactions, we decided to focus our study on the function of pyoverdine and pyochelin secreted by *P. aeruginosa* on the modulation of *Scedosporium/Lomentospora* growth. For the 8737A, 22959, 22960 and 23007 *P. aeruginosa* strains, the concentration of pyoverdine and pyochelin in the extracellular milieu increased significantly in the presence of *Scedosporium* and *Lomentospora* species compared to bacterial pure cultures; this profile was not detected for 8737B and 23008 strains of *P. aeruginosa*. Variations in the production of virulence factors among CF strains is expected, since CF environment leads to divergence in subpopulations of *P. aeruginosa* due to the nutritional composition, reduced dispersal resulting from the thick mucus associated with *CFTR* gene defect, and isolation of strains from different lung regions [44,45]. In conformity, one-third of *P. aeruginosa* isolates from CF lost the ability to produce pyoverdine [46]. Most importantly, the increased production of *P. aeruginosa* metabolites after co-culture with fungal cells is usually induced by microbial competition for resources [20,25,36,42,47]. 

In order to evaluate the conditions in which fungal cells induce the secretion of siderophores by *P. aeruginosa* cells, a series of experiments were performed in the present study to verify: (i) the effect of direct microbial contact, (ii) the production of these molecules in the presence of metabolically inactive fungal cells, and (iii) the microbial interaction under different iron concentrations. The induction of siderophores production occurred even when the microbial cells were physically separated but sharing the same culture medium, reinforcing that the increase in siderophore production is due to the competition for the iron available in SCFM and not due to the direct interaction between surface molecules of fungi and bacteria. Accordingly, the growth inhibition of *S. aurantiacum, S. apiospermum* and *S. boydii* during indirect interaction with *P. aeruginosa* was previously described [15,28]. As siderophores are secondary metabolites that are produced when iron is not readily available [48], we revealed that the induction of siderophores production occurred mainly during the interaction with metabolically active fungal cells and in iron-limiting conditions. Collectively, these results corroborate the hypothesis that induction of siderophores production in *P. aeruginosa* is due to the microbial dispute for available iron. 

The direct effect of *P. aeruginosa* secreted molecules on *Scedosporium/Lomentospora* was subsequently evaluated. The inhibition of fungal growth by *P. aeruginosa* secreted molecules can occur in several ways: (a) germination blockage through iron denial to fungal cells by pyoverdine and pyochelin, (b) depletion of zinc and copper also caused by bacterial siderophores, (c) induction of ROS and RNS by bacterial phenazines, and (d) inhibition of fungal ꞵ-1,3-glucan synthase by dirhamnolipids [17,25,49,50]. Supernatants of *P. aeruginosa* were able to inhibit the biomass formation by *Scedosporium/Lomentospora* species; however, in 24 h mature fungal biofilms, the conditioned supernatant had no effect. Similarly, *P. aeruginosa* inhibits *A. fumigatus* conidia, but has minor effects on germinated conidia and mature biofilms [34,51]. The differences in inhibitory capacity of *P. aeruginosa* to conidia and hyphae is attributed to differences on the metabolic activity of both fungal morphotypes: conidial cells present an intense metabolic activity—necessary for cell differentiation, for example—whereas in the hyphae, the activity is reduced, being restricted to the apical region [52]. Moreover, differences on the architecture of conidial and hyphal cell surfaces could interfere with *P. aeruginosa* molecules permeability [51,52]. In our work, the conditioned supernatants obtained in the presence of fungal cells presented a higher inhibitory effect than conditioned supernatants from *P. aeruginosa* pure cultures (particularly strains 8737A, 22959, 22960 and 23007), which correlates with the increase in siderophores concentration. 

To reinforce the role of pyoverdine and pyochelin in *Scedosporium/Lomentospora* growth inhibition, we tested 5-FC, an antifungal drug that presents a secondary activity as an inhibitor of pyoverdine production [18,26,27]. 5-FC inhibits the iron-starvation σ-factor PvdS expression, which is essential to transcription of pyoverdine biosynthetic pathway genes [26,53]. As expected, the conditioned supernatants of *P. aeruginosa* cells obtained in the presence of 5-FC had a significantly lower concentration of siderophores and a lesser capacity to inhibit the growth of *Scedosporium/Lomentospora* species. Despite the inhibition of more than 85% on siderophores production, the inhibition of fungal growth by the conditioned supernatant obtained in the presence of 5-FC was only slightly lower compared to control supernatants, suggesting that other secreted molecules can also contribute to this inhibition phenomenon. In accordance, pyocyanin and *cis*-11-methyl-2-dodecenoic acid (DSF) have been demonstrated to have the ability to inhibit the *Scedosporium* conidial germination [14]. Herein, we showed the role of pyoverdine and pyochelin in *Scedosporium/Lomentospora* inhibition during the interaction with *P. aeruginosa*. However, in a study conducted by Le Govic and co-workers [54], it was demonstrated the iron acquisition from pyoverdine by the siderophore *N^α^*-methylcoprogen B produced by *S. apiospermum.* Further studies are necessary in order to better understand the different phenomena detected in both researches, but we can raise some hypotheses: (i) the concentration of pyoverdine could interfere with the outcome of fungal growth, since this bioactive molecule can present both inductive or inhibitory effects on *A. fumigatus* depending on its concentration [49]; (ii) in the Le Govic and co-workers study [54], the effect of pyoverdine was evaluated with the purified molecule, whereas we utilized a pool of molecules secreted by *P. aeruginosa,* so the function of one molecule could interfere with the activity of another; and (iii) the culture media (SCFM versus potato dextrose agar) utilized in each work, besides the difference between the growth and molecule diffusion in liquid and solid media, could also interfere in pyoverdine production. Accordingly, in a study performed with *A. fumigatus*, it was determined that the major antifungal molecules produced by *P. aeruginosa* in liquid medium (siderophores) differ from those produced on solid media (rhamnolipids and elastase) [55].

## 5. Conclusions

The present study adds new findings on the complex and multimodal interaction events between clinically relevant bacteria (e.g., *P. aeruginosa*) and fungi (*Scedosporium/Lomentospora* species) in the CF context. In this scenario, we started to explore the role of environmental iron concentration in the interaction between *P. aeruginosa* and *Scedosporium/Lomentospora* species in a CF mimic environment. In summary, we demonstrated the induction of bacterial siderophores production due to the fungal presence, which leads to iron denial for fungal cells and consequently fungal growth inhibition. Further studies are necessary in order to understand how *Scedosporium/Lomentospora* species respond to the increase in bacterial secondary metabolites, as well how the fungal-bacterial interaction influences the pathogenicity and the antimicrobial treatment in colonized CF patients. 

## Figures and Tables

**Table 1:** Data about *P. aeruginosa* strains used in this study.

CF Codes	Strain Codes	Bacterial Phenotypes
Patient 1	8737A	Mucoid
Patient 1	8737B	Non-mucoid
Patient 2	22959	Mucoid
Patient 2	22960	Non-mucoid
Patient 3	23007	Mucoid
Patient 3	23008	Non-mucoid

## Data Availability

Not applicable.
